# Improvements in SOD mimic AEOL-10150, a potent broad-spectrum antioxidant

**DOI:** 10.1186/s40779-018-0176-3

**Published:** 2018-09-06

**Authors:** Xiao-rui Zhang, Wen-xia Zhou, Yong-xiang Zhang

**Affiliations:** State Key Laboratory of Toxicology and Medical Countermeasures, Beijing Institute of Pharmacology and Toxicology, Beijing, 100850 China

**Keywords:** Catalytic antioxidant, Metalloporphyrins, Chemical warfare agent, Radiation damage, Pharmacodynamics, Pharmacokinetics

## Abstract

AEOL-10150 is a broad-spectrum metalloporphyrin superoxidase dismutase (SOD) mimic specifically designed to neutralize reactive oxygen and nitrogen species. Research has shown that AEOL-10150 is a potent medical countermeasure against national security threats including sulfur mustard (SM), nerve agent exposure and radiation pneumonitis following a radiological/nuclear incident sufficient to cause acute radiation syndrome (ARS). AEOL-10150 performed well in animal safety studies, and two completed phase 1 safety studies in patients demonstrated that the drug was safe and well tolerated, indicating that AEOL-10150 has potential as a new catalytic antioxidant drug. In this article, we review improvements in AEOL-10150 in preclinical pharmacodynamic studies, especially regarding anti-SM, chlorine gas and radiation exposure studies.

## Background

Superoxide dismutase (SOD) is a cytoprotective enzyme [1]. The SOD pioneer Fridovich first synthesized pyridine quaternary ammonium water-soluble manganoporphyrin to mimic the Mn-SOD catalytic site [2, 3], after which a series of compounds were reported. AEOL is a type of metalloporphyrin catalytic antioxidant developed by US Aeolus pharmaceuticals that possesses SOD- and catalase-like activity. As a leading AEOL compound, AEOL-10150 was studied as a medical treatment against acute radiation-induced lung injury using the biomedical advanced research and development authority’s $118 million 5-year grant. In follow-up research, Aeolus pharmaceuticals uncovered additional AEOL-10150 biological activities for treating sulfur mustard (SM), chlorine gas and nerve agent exposure and acquired an National Institutes of Health (NIH) grant to develop AEOL-10150 as an anti-chemical warfare agent [4]. If the project is successful, AEOL-10150 would be stocked in the U.S. Strategic National Stockpile (SNS).

**Fig. 1:**
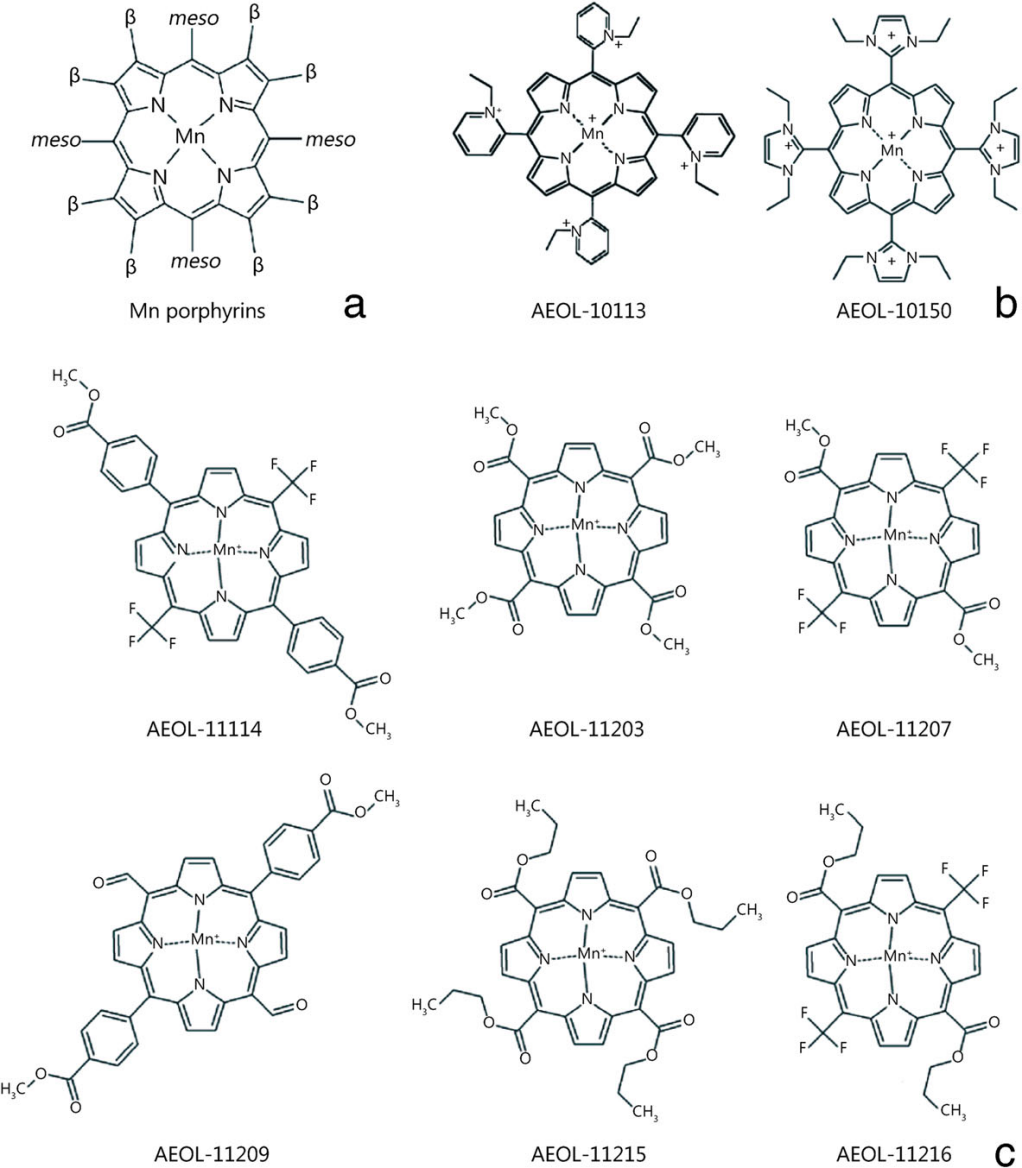
The structure of AEOL compounds. **a**. Structural scheme of the Mn porphyrins of SOD mimics; **b**. Water-soluble compounds of Mn porphyrins; **c**. Lipid-soluble compounds of Mn porphyrins

AEOL-10150 is also known as MnTDE-2-ImP5^+^, with the molecular formula C48-H56-Mn-N12.5-Cl and a molecular weight of 1033.257. Structurally, SOD mimics are characterized by a modified alkylated pyridyl quaternary ammonium group at the meta position of the porphyrin ring. Coordination manganese ions are generally + 3 charges (Fig. 1a). Studies show that the position of the N atom on meso-pyridine significantly affects manganese porphyrin activity and that the more active the pyridine N is, the closer it is to the metal catalyst center [5]. Among the AEOL series compounds, the activities of water-soluble AEOL-10150 compounds deserve attention. Recent reports have shown that AEOL-1114 and AEOL-11203 exhibit good lipophilicity and, upon oral administration, can pass through the blood cerebrospinal fluid barrier (Fig. 1b, c) [6]. In addition, Aeolus improved the production of AEOL-10150 in terms of drug stability, reducing costs by 90%, and confirmed that new synthetic AEOL-10150 is stable at room temperature and under refrigerated conditions up to 24 months; stability testing will continue to 60 months [7].

Aeolus pharmaceuticals announced that AEOL-10150 achieved excellent safety results in both animal and human experiments. One of the two clinical studies of 50 patients (at least 39 patients with amyotrophic lateral sclerosis (ALS)) confirmed that AEOL-10150 did not cause serious side effects and was well tolerated, although skin spots were caused by discoloration and continuous infusion at the injection site [8]. AEOL-10150 was approved by the Food and Drug Administration (FDA, fast track designation) for treating patients with lung acute radiation syndrome (ARS) (Table 1). The new and old formulations of AEOL-10150 have been reported to exhibit similar toxicity and pharmacokinetic characteristics, and more importantly, the new formulation of AEOL-10150 exhibits less irritation and has higher bioavailability. Based on prepharmacy, animal-level pharmacodynamics, toxicology, and pharmacokinetics studies, Aeolus announced the launch of a new AEOL-10150 formulation for human phase I clinical trials in healthy volunteers and/or to test its potential to treat pulmonary fibrosis patients by evaluating its safety, tolerance and pharmacokinetics [4]. In this article, we focus on AEOL-10150 advances in preclinical pharmacodynamics, especially anti-SM, chlorine gas and radiation exposure studies.

**Table 1:** Improvements in AEOL-10150 regarding antiradiation diseases released by Aeolus Pharmaceuticals

Time (M/D/Y)	Contents
04/15/2008	AEOL-10150 protects lungs against fractionated radiation damage and inhibits angiogenesis and inflammation
07/06/2009	AEOL-10150 significantly improves survival in mice exposed to radiation when administered after exposure
09/08/2009	Initiates study of AEOL-10150 as a treatment against radiation exposure in nonhuman primates
11/17/2009	Duke University initiates study of AEOL-10150 to observe the compound’s activity protecting healthy tissue in mice receiving chemotherapy and radiation for non-small-cell lung cancer
04/12/2010	Proclaims the initiation of a second study of AEOL-10150 as a medical treatment against GI acute radiation syndrome funded by the NIH’s National Institute for Allergy and Infectious Diseases
11/02/2010	AEOL-10150 improves survival in nonhuman primates exposed to lethal doses of radiation
02/08/2012	AEOL-10150 protects lung tissue from radiation by regulating PTEN levels and inhibiting oxidative stress and apoptosis
06/01/2012	AEOL-10150 alleviates lung damage after Neupogen treatment following radiation exposure
09/17/2013	BARDA exercises $6.0 mm in additional contract funding to develop AEOL-10150 as countermeasure against acute radiation syndrome
10/29/2013	AEOl-10,150 significantly increases survival and protects lungs in mice exposed to lethal radiation
08/20/2014	Aeolus files an investigational new drug application with the FDA to enable the initiation of human safety studies for the development of AEOL-10150 as a medical treatment against lung damage from radiation exposure
09/04/2014	Proclaims positive results from a study showing AEOL-10150 doubles the survival rate following lung damage caused by acute radiation exposure
05/04/2015	Aeolus proclaims complete results from a successful study showing AEOL-10150 doubles survival rate following lung damage from acute radiation exposure
06/26/2015	BARDA exercises $3 mm in additional contract funding for the development of AEOL −10,150 as a countermeasure to acute radiation syndrome
06/08/2017	FDA fast track designation granted to AEOL-10150 for treatment of patients with lung acute radiation syndrome following a radiological or nuclear event

### Anti-chemical warfare agent exposure by AEOL-10150

#### Anti-sulfur mustard exposure

AEOL-10150 has broad-spectrum antioxidant activity, including the removal of O_2_^−^, H_2_O_2_, ONOO^−^, NO and lipid peroxides [9–11]. The indications mainly focus on treating SM, nerve agents and other chemical warfare agents (Table 2), and radiation damage (Table 1) and other oxidative stress-related diseases. SM is a vesicating and alkylating chemical warfare agent with extremely high activity for which there is still no antidote. The SM analog 2-chloroethyl ethyl sulfide (CEES) has been used to study the mechanisms of SM intoxication and to screen for antidotes. One study showed that post-treatment with AEOL-10150 reduces CEES-induced lung injury in rats exposed to 5% CEES for 15 min with a nose-only inhalation system. The rats were then injected with 5 mg/kg AEOL-10150 subcutaneously 1 h and 9 h after CEES exposure to observe antitoxic effects. The results showed that lactate dehydrogenase, protein, IgM, erythrocyte and neutrophil levels significantly increased in the bronchoalveolar lavage fluid of rats 18 h after exposure, and AEOL-10150 treatment significantly inhibited these affects. This study also found that myeloperoxidase (MPO) activity significantly increased in the lungs of rats after inhalation and was significantly reduced after AEOL-10150 administration. In addition, the oxidative stress markers 8-OHdG and 4-hydroxynonenal (4-HNE) also increased significantly after exposure and significantly decreased after AEOL-10150 treatment. This study showed that CEES inhalation clearly causes lung injury, inflammation and oxidative stress that can be significantly antagonized by AEOL-10150 [12]. Studies have found that CEES inhalation leads to extensive olfactory epithelium (OE) damage. AEOL-10150 reduces OE damage both subcutaneously (5 mg/kg) and combined with intranasal administration (50 μmol/L) [13], indicating that AEOL-10150 is a countermeasure candidate for SM-induced lung injury.

**Table 2:** Improvements in AEOL-10150 in terms of use as an anti-chemical warfare agent released by Aeolus Pharmaceuticals

Time (M/D/Y)	Contents
11/07/2007	AEOL-10150 protects SM-induced lung damage
04/23/2008	AEOL-10150 significantly protects against SM-exposure-induced lung and skin damage
02/09/2009	NIH Counter ACT program begins assessing treatment with Aeolus pharmaceuticals’ AEOL-10150 against SM gas damage
10/07/2009	AEOL-10150 protects lungs against SM damage in animal research
11/04/2009	AEOL-10150 protects lungs against Cl_2_ exposure in animal research
06/29/2010	AEOL-10150 significantly protects skin in preclinical SM damage studies
06/30/2010	AEOL-10150 protects lungs against SM exposure in animal research
10/10/2011	NIH grant of $12.7 million awarded to continue development of AEOL-10150 as a countermeasure to Cl_2_ and SM damage
10/24/2011	Aeolus pharmaceuticals announces NIH award to develop AEOL-10150 as a countermeasure to nerve agent exposure
07/30/2012	AEOL-10150 announced to eliminate oxidative stress and nerve damage following exposure to nerve agent
06/26/2013	AEOL-10150 significantly ameliorates survival in animals exposed to SM
07/02/2013	AEOL-10150 significantly ameliorates survival from nitrogen mustard exposure to skin in animals
09/11/2013	AEOL-10150 is the subject of a $4.3 million US government award defining countermeasures to nerve agents; data show that the drug significantly ameliorates survival in animals following exposure to pilocarpine, a nerve agent surrogate for soman and sarin gas
06/18/2014	Aeolus proclaims the publication of data demonstrating the efficacy of AEOL-10150 in an animal CEES model of SM skin damage
07/01/2014	Aeolus proclaims additional data demonstrating efficacy of AEOL-10150 as a medical treatment against exposure to nerve agents, SM and nitrogen mustard gas
03/31/2015	Aeolus receives a notice of allowance from the Israeli patent office for the use of AEOL-10150 and other Aeolus compounds to treat damage from exposure to SM
09/06/2016	Aeolus proclaims positive data demonstrating the efficacy of AEOL-10150 as a medical treatment against SM
09/19/2016	Aeolus proclaims publication of additional data showing the efficacy of AEOL-10150 in SM exposure in the journal *Toxicological Sciences*

Studies have also monitored the effects of AEOL-10150 on CEES-induced skin damage. Administration of AEOL-10150 (50 μmol/L) 1 h after CEES exposure significantly reversed the CEES-induced necrosis of murine epidermal JB6 cells, human HaCaT cells and normal human epidermal keratinocytes and reduced DNA damage. AEOL-10150 also drastically ameliorated the cytoplasmic and mitochondrial active oxygen free radicals in JB6 and HaCaT cells to reduce skin damage. This study indicated that AEOL-10150 has potential therapeutic efficacy against CEES-mediated skin lesions and supports AEOL-10150 as an effective medical countermeasure against skin lesions caused by SM [14].

Recent research studied the effect of AEOL-10150 on SM exposure. Adult male rats were intubated, followed by SM (1.4 mg/kg) exposure, which exhibits an LD50 at approximately 24 h. Rats were injected subcutaneously with sterile PBS or AEOL-10150 (subcutaneous injection, every 4 h, 5 mg/kg) beginning 1 h post-SM exposure. Notably, catalytic antioxidant treatment improved survival after SM inhalation in a dose-dependent manner, and the survival rate of animals in the administration group was as high as 52% at 48 h after SM exposure. The drug also maintained a 72 h response when treatment was terminated 48 h after SM exposure. The test results showed that the oxygen saturation of treated animals increased by 10%, and the clinical score reduced by 57%. Tissue analysis revealed a 69% reduction in airway tube formation in treated animals within 48 h of SM exposure. To determine the effect of drug treatment at peak injury, changes in oxidative stress markers were observed 24 h after SM exposure. AEOL-10150 significantly reduced SM-induced lung injury by reducing lung lipid peroxidation and inhibiting the release of multiple proinflammatory cytokines, suggesting that catalytic antioxidants are effective measures of SM inhalation injury [15]. The above results indicate that AEOL-10150 might be an effective anti-SM treatment agent. In future research, AEOL-10150 anti-mustard gas research should elucidate the molecular mechanisms and targets of drug efficacy. Understanding these mechanisms would help elucidate the mechanism of mustard gas poisoning and significantly drive the development of anti-SM drugs.

#### Antichlorine-induced lung injury

Chlorine (Cl_2_) is a typical asphyxial chemical reagent, and rescue treatment after Cl_2_ exposure is under intense research [16, 17]. Mice were exposed to 100 ppm Cl_2_ gas for 5 min and then divided into four groups: the chlorine only exposure group, treated with AEOL-10150 1 h and 9 h after chlorine exposure group; the AEOL-10150 only group; and the control group. An experimental small animal ventilator was used to measure airway responsiveness to aerosolized methacholine (6.25–50 mg/ml) 24 h after Cl_2_ gas exposure, and bronchial alveolar lavage (BAL) was used to assess airway inflammation and protein. The 4-hydroxynonenal in whole lung tissue was tested. Lung tissue from different groups of animals was collected 72 h after Cl_2_ exposure to measure epithelial cell proliferation. Compared to other groups, mice exposed to Cl_2_ showed significantly higher airway resistance in response to methacholine challenge. AEOL-10150 significantly attenuated the increase in neutrophils and macrophages in the BAL of Cl_2_-exposed mice. AEOL-10150 significantly inhibited the Cl_2_-induced increase in BAL fluid protein and also attenuated the Cl_2_-induced increase in epithelial cell proliferation. AEOL-10150 significantly reduced 4-HNE levels after Cl_2_ exposure. The above results indicate that AEOL-10150 is an effective treatment for Cl_2_-induced airway hyperresponsiveness, airway inflammation, airway epithelial cell proliferation and oxidative stress [18]. The above results indicate that AEOL-10150 may be a potent inhibitor of Cl_2_ damage including airway hyperresponsiveness, airway inflammation, oxidative stress and epithelial cell proliferation. In future studies, AEOL-10150 anti-Cl_2_ research should further elucidate the targets for drug efficacy to elucidate the mechanism of Cl_2_ poisoning and promote the success rate of research and development of anti- Cl_2_ drugs.

### Anti-radiation exposure of AEOL-10150

Radiation damage is closely related to free radical release [19–21]. Studies have shown that chronic administration of the SOD mimic AEOL-10150 has a protective effect on radiation-induced lung injury, whereas low-dose or short-term AEOL-10150 treatment has no effect on radiation-induced lung injury [22, 23]. Early studies have found that the chronic sustained expression of extracellular SOD (EC-SOD) protects lungs from radiotherapy-induced injury. Therefore, AEOL-10150 is used to treat lung injury after radiotherapy [24]. The results showed that the respiratory rate of Fisher-344 rats increased 8 weeks after radiotherapy (28 Gy), reaching a peak at 18 weeks. Low-dose AEOL-10150 via osmotic pump (1 mg/(kg.d)) conferred no protection against radiotherapy-induced injury. Compared with the model group, the experimental animals of the medium- and high-dose AEOL-10150 administration groups (10 and 30 mg/(kg.d)) showed significantly decreased respiratory rates. Immunohistochemistry showed that the number of macrophages in the middle- and high-dose groups was significantly reduced [25].

Recently, a study examined the effect of AEOL-10150 on lethal lung radiation injury (10.74 Gy, 0.80 ± 0.05 Gy/min) of rhesus macaque lungs. The researchers designed three treatment options, including radiotherapy day 1-day 28, day 1-day 60 or a divided regimen day 1-day 28 plus day 60-day 88. The results showed that only the day 1-day 60 administration schedule improved survival from 25 to 50%, with an average survival time of decedents and latency to a non-sedated respiratory rate of > 60 or > 80 breaths/min; furthermore, lung injury was eliminated. This treatment did not improve the incidence of pneumonitis or the severity of fibrosis. The Kaplan-Meier survival curves indicated that treatment efficacy could be strengthened by lengthening treatment time to 90 days or longer after lung irradiation [26]. Another nonhuman primate model showed that treatment with AEOL-10150 (5 mg/kg) for 1 month resulted in 28.6% survival following exposure to a radiation dose of 11.5 Gy that proved to be 100% fatal in control animals. The AEOL-10150-treated group required less dexamethasone support during the survival phase of the study. Compared with the control cohort, the AEOL-10150 treatment group showed lower plasma TGF-β_1_ levels [27].

In addition, the ability of AEOL-10150 to reduce the severity of long-term lung injury caused by fractionated radiotherapy (RT) has been assessed. The experimental rats were randomly divided into 5 groups: RT + 2.5 mg/kg AEOL-10150, RT + 5 mg/kg AEOL-10150, AEOL-10150 2.5 mg/kg group, AEOL-10150 5 mg/kg group and RT. The right hemithorax received five 8 Gy fractions of RT. After 26 weeks, lung tissue showed that lung injury and collagen deposition in the 5 mg/kg AEOL-10150 treatment group significantly decreased compared with that in the RT alone group. The research also showed significant reduction in hypoxia inducible factor-1α (HIF1α), Vascular endothelial growth factor, CD-31, Fibroblast growth factor receptor 2 IIIc (ED-1), 8-hydroxy-2 deoxyguanosine (8-OHdG), 3-nitrotyrosine, transforming growth factor β_1_ (TGFβ_1_), Small mother against decapentaplegic 3 (Smad3), and p-Smad2/3 in animals in the RT+ 5 mg/kg AEOL-10150 group. Although the administration of AEOL-10150 (5 mg /kg, bid) during and after RT had a significant protective effect on lung injury, the low-dose AEOL-10150 (2.5 mg/kg bid) administration group exhibited no significant effect [28, 29]. Related research additionally identified 44 genes associated with oxidative stress, cell growth, inflammation, extracellular matrix synthesis metabolism and apoptosis that increased after radiation. In the animals treated with AEOL-10150, most genes with increased expression attenuated, indicating that some hypoxia-related genes effectively regulated after irradiation [30]. The anti-radiation effect of AEOL-10150 may partially relate to its apoptotic inhibition. Normal lung tissues several weeks after RT showed I and II type pneumocytes and endothelial cell apoptosis. Furthermore, apoptosis-related signaling pathways revealed that PTEN expression increased, the PI3K/AKT signal was inhibited, and p53 and Bax protein expression levels increased, which might be related to radiation-induced increases in TGF-β_1_, NADPH oxidase 4 (Nox4) and oxidative stress. After 2 h of irradiation, AEOL-10150 (20 mg/(kg.d), once every other day for 4 weeks) restored PTEN and the downstream proapoptotic signal to normal levels and reduced apoptosis [31]. A study also found that AEOL-10150 had a positive effect on the incidence and duration of gastrointestinal repair and radiation-induced oral mucositis after RT [32]. Although the above studies evaluated the effects of AEOL-10150, compared with other antioxidants, against radiation damage and elucidated some of the molecular mechanisms, the main target of AEOL-10150 and the characteristics of its effects remain unclear.

### Other pharmacodynamics of AEOL-10150

#### Lung injury protection

Smoking leads to respiratory inflammation and epithelial damage, and smoke itself or the persistent inflammation generated by free radicals can be important driving forces [33]. For example, the number of cells recovered from bronchoalveolar lavage was significantly elevated after tobacco smoke exposure of either 2 days or 8 weeks (6 h/d, 3 d/week). AEOL-10150 significantly reduced bronchoalveolar lavage cell number in tobacco smoke-treated rats. Obvious decreases in neutrophil numbers were observed at 2 d and in macrophages after 8 weeks. Lymphocytes were significantly decreased by AEOL-10150 at both time points. After 8 weeks of exposure to tobacco smoke, the number of squamous metaplasia treated with AEOL-10150 decreased from 12% of the total airway epithelial area to 2%. The results show that AEOL-10150 has a protective effect on smoking-induced bronchial lung injury [34]. In addition, studies have found that AEOL-10150 also reduces the remodeling of smoking-induced lung tissue [35]. The protective effect of AEOL-10150 on smoking-induced lung injury may also be associated with the role of AEOL-10150 in controlling Nuclear factor κB (NF-κB) activation, affecting histone modification and expression of inflammatory genes [36, 37]. As oxidative stress and chronic inflammation are also important pathologic features of chronic obstructive pulmonary disease (COPD) [38, 39], it was concluded that AEOL-10150 might be beneficial for the treatment of COPD.

#### Cerebral ischemia-reperfusion injury protection

Oxidative stress is an important cause of cerebral ischemia-reperfusion injury [40]. The effect of AEOL-10150 on middle cerebral artery occlusion (MCAO) was observed. Rats were intraperitoneally injected with AEOL-10150 for 90 min after reperfusion for 90 min. The results showed that AEOL-10150 significantly reduced infarct size (35%) and neuronal loss, and in the ischemic cerebral hemisphere, AEOL-10150 uptake was time and dose-dependent. AEOL-10150 inhibited lactate dehydrogenase release in a dose-dependent manner in a neuronal/glia mixed culture system deprived of oxygen and glucose for 2 h and selectively retained aconitase at concentrations consistent with neuroprotection as assessed by an in vivo enzyme activity. The results of this study indicate that AEOL-10150 has a wide therapeutic window range and neuroprotective activity [41]. In addition, AEOL-10150 intravenous administration to treat ischemia-reperfusion also has a significant therapeutic effect. Studies have shown that AEOL-10150 administration decreased the cerebral infarction area by 40% and significantly improved nervous system function in C57BL/6 J mice subjected to 60 min of MCAO with the pericranial temperature controlled at 37 °C [42]. Similarly, C57BL/6 J mice were treated with AEOL-10150 intravenously for 5 min on the right MCAO and 5 min after reperfusion. Stress and inflammatory response-related gene expression changed significantly, including HSP (heat shock protein), IL-6, and MIP-2 (macrophage inflammatory protein-2). AEOL-10150 treatment is limited to inhibiting the expression of inflammatory genes. [43]. These results suggest that AEOL-10150 can effectively reduce ischemic brain damage if administered intravenously at an early stage of reperfusion after cerebral ischemia, and its mechanism may be partly related to a reduced inflammatory response.

#### Spinal cord injury protection

Spinal cord injury is closely related to ROS [44]. AEOL-10150 also has protective effects on mouse spinal cord injury. Studies have evaluated the efficacy of AEOL-10150 in spinal cord compression (SCC) mice and showed that AEOL-10150 (0.5 mg/kg rapid injection and then 1 mg/(kg·h) continuous administration for 24 h) given intravenously 5 min after injury in SCC model mice or treatment with methylprednisolone (30 mg/kg injection, and then 5.4 mg/(kg·h) for 24 h) had no significant effect on the rotation test or the total lesion score. However, if intradermal administration was used, AEOL-10150 (2.5 or 5.0 mg) improved both the rotation test and total damage score compared with the control group. This study shows that the therapeutic effect of SCC on AEOL-10150 depends on its concentration in the central nervous system [45, 46].

#### Anti-amyotrophic lateral sclerosis

It is well known that mice overexpressing the human Cu, Zn SOD1 mutant G93A gradually develop delayed and progressive motor neuron diseases similar to human ALS [47]. Studies have shown that if AEOL-10150 (intraperitoneal injection) at an initial dose of 5.0 mg/kg and a maintenance dose of 2.5 mg/(kg·d) is started at the onset of symptoms, the animal survival period can be effectively prolonged 3.0-fold. Spinal cord immunohistochemistry results showed that AEOL-10150 treatment induced expression of the SMI-32 protein and that the protein in motor neurons has a high level of expression. The results showed that AEOL-10150-treated spinal cord motor neurons had better-preserved architecture with fewer astroglial cells (glial fibrillary acidic protein) and significantly less nitrotyrosine and malondialdehyde. These results demonstrate that AEOL-10150 has a definite therapeutic effect on ALS onset [48]. In addition, the study found that the use of AEOL-10150 alone or the histone deacetylase inhibitor phenylbutyric acid (PBA) significantly enhanced motor function and prolonged survival by 11% and 13%, respectively. The combined application of PBA and AEOL-10150 significantly extended survival by 19% [49]. Drugs evaluated based on the SOD1 mouse model failed to identify drugs effective for human ALS treatment, leading to questions regarding whether these preclinical data could be used for human disease research. Subgroup analysis indicated that COX-2 inhibitors with anti-inflammatory activity, the antibiotic minocycline, and the antioxidant creatine or AEOL-10150 are the most promising prophylactic and therapeutic agents for patients with familial ALS [50].

#### Islet cell protection

For many reasons, a significant reduction in the amount of transplantable islet cells occurs [51]. The researchers found that adding SOD mimetics significantly increased islet cell survival. The function and phenotypic characteristics of islets treated with SOD were preserved, and even transplanted peripheral cells of islet tissue restored function to diabetic mice. Further inhibition of islet cell loss can be achieved if SOD mimetics are added earlier. [52]. It has been further found that AEOL-10150 significantly reduces NF-κB binding to DNA, inhibiting the release of inflammatory cytokines and chemokines as well as the activation of poly ADP-ribose polymerase (PARP) polymerase, resulting in greater survival and much better insulin release [53]. A similar study found that the best dosage able to fully counteract mechanical stress was 100 μmol/L. At dosages ≥150 μmol/L, the compound was toxic for islet cells. In contrast, AEOL-10150 offers no protection against rIL-1β-induced islet cell chemical stress damage [54]. These results suggest that antioxidants are beneficial for reducing islet cell damage and have a significant effect on islet transplantation.

#### Effects on the cardiovascular system

EC-SOD has protective effects on myocardial ischemia, pulmonary inflammation and fibrosis [55]. Studies have shown that, compared with wild-type mice, EC-SOD knockout mice have thinner left ventricular walls, increased end-diastolic volume, increased cardiomyocyte apoptosis, and left ventricular fibrosis and inflammatory cell infiltration. Doxorubicin can cause oxidative stress in the heart. Doxorubicin can further elevate cardiomyocyte apoptosis and left ventricular fibrosis and inflammatory cell infiltration in EC-SOD knockout mice, whereas AEOL-10150 eliminates cardiac dysfunction and myocardial fibrosis in doxorubicin-induced wild-type mice and EC-SOD knockout mice [56]. Endothelin-1 (ET-1) is a vasoconstrictor and mitogen peptide that regulates and maintains blood pressure [57, 58]. Because oxidative stress activates the vasoconstrictor system, ET-1 is closely linked to pulmonary and cardiovascular diseases, including congestive heart failure, atherosclerosis, and hypertension [59, 60]. Therefore, SOD mimics have been proposed to be used to observe the role of ET-1 in the endothelin system. Subcutaneous injection of AEOL-10150 (2 or 5 mg/kg) after 2 h and 24 h has been reported to significantly reduce Fischer-344 male rat isoeperolane (− 25%) and 3-nitrotyrosine (− 50%), and the decreased oxidative stress markers were consistent with decreased plasma ET-1 (− 50%) and the related endothelin ET-3 (− 10%) within 24 h. Although PreproET-1 and endothelin-1 (ECE-1) mRNA levels did not change in the lungs, there was a significant increase in preproET-1 and ECE-1 mRNA levels (10–25%). Lung ET-1 clearance receptor and vasoconstriction-signal ETA receptor mRNA levels were significantly decreased; iNOS severely strongly initially but persistently declined by 40% in 24 h. The results suggest that AEOL-10150 regulates the endothelin/nitric oxide system by inhibiting endogenous ROS and RNS [61].

#### Immune regulation

Manganese porphyrins are considered the most active free radical scavengers, in which Mn is positively charged and reacts with negatively charged free radicals such as O_2_^−^ and ONOO^−^. Some researchers believe that the antioxidant-regulated redox reaction may be beneficial for the control of inflammation-related diseases [62]. To validate this hypothesis, researchers have observed the effects of AEOL-10150 on LPS-induced secretions of the proinflammatory cytokines TNF-α, IL-1β and free radical NO_2_^−^ and O_2_^−^. Antioxidants inhibited free radical and proinflammatory cytokine production and were associated with the inhibition of NF-κB binding to DNA, independent of MAPK signaling pathways [63]. Compared with pulmonary hemorrhage in wild-type mice, in EC-SOD-deficient mice 1 h after pulmonary hemorrhage neutrophil aggregation, myeloperoxidase activity was increased 3.9 times, and NF-κB and lipid peroxidation enzyme activity was increased by 1.5 times. Prophylactic administration of AEOL-10150 significantly reduced NF-kB and lipid peroxidase activity in both wild-type and EC-SOD-deficient mice [64]. In addition, manganese porphyrin has been reported to inhibit transcription factors HIF-1α, AP-1 and SP-1 [65]. The above studies suggest that catalytic antioxidants can be used for the treatment of inflammatory-related diseases.

#### Antitumor

Antioxidants have protective effects on radiation damage in normal tissues, but the effects on radiotherapy-receiving tumor cells are unclear [66, 67]. Studies have examined the effect of AEOL-10150 on tumor cells in tumor-bearing mice with RM-9 prostate cancer undergoing radiation therapy (10 Gy). The results showed that the tumor volume reduction was more pronounced in the AEOL-10150 combined radiotherapy group. The level of HIF-1α in tumor cells was significantly higher than that in the control group (*P* < 0.05). However, the level of HIF-1α in tumor cells in the treatment group significantly decreased, the secretion of TNF-α decreased, and the secretion of IL-4 was significantly increased. These results suggest that AEOL-10150 has no protective effect on RM-9 prostate tumors but rather has radiotherapy enhancement, which may be related to its inhibition of radiotherapy-induced release of HIF-1α and changes in cytokine distribution [68].

Another hypothesis is that the antitumor effect of AEOL-10150 may be related to its pro-oxidative activity. SOD-deficient aerobic *E. coli* is a simple system that demonstrates that manganese porphyrins can shift from antioxidant activity to pro-oxidative activity. In this system, AEOL-10113 can catalyze the production of hydrogen peroxide in the presence of ascorbic acid and lead to oxidative damage and inhibition of growth in wild-type and SOD-deficient *E. coli*. If catalase is added to the medium, the above effects can be reversed, indicating that hydrogen peroxide is an important signaling molecule that causes growth inhibition. Further experiments using oxyR- and soxRS-deficient *E. coli* revealed that oxidative stress induced by the manganese porphyrin/ascorbic acid system activates the oxyR modulator, which in turn activates the antioxidant system, such as the induction of catalases and peroxidases enzyme production. As expected, *E. coli* does not upregulate the antioxidant system to remove hydrogen peroxide when hydrogen peroxide is added [69]. In addition, the catalytic antioxidant itself has an oxidative effect on NF-kB, thereby inhibiting NF-kB binding to DNA, which can be reversed by the reducing agent DTT [64]. The above studies show that manganese porphyrins do have oxidative activity. Based on the characteristics of manganese porphyrins, it can be speculated that manganese porphyrin combined with ascorbic acid in vitro has anticancer activity, and when combined with chemotherapy drugs in vivo, manganese porphyrin has a therapeutic effect on lymphoma, which may be caused by its promotion of oxidative activity. Notably, whether manganese porphyrins play an antioxidant or oxidative effect in vivo depends on the level of intracellular free radicals and endogenous antioxidants, oxygen utilization, superoxide anion and superoxide removal system ratio, manganese porphyrins themselves and their redox capacity and their position in the cell and bioavailability.

## Conclusions

Preclinical studies have shown that AEOL-10150 has a significant antitoxic effect on chemical agents such as SM and nerve agents. AEOL-10150 is also an efficacious countermeasure to radiation damage. In addition, AEOL-10150 has the potential to be developed as a new drug against tumors, hemorrhagic reperfusion injury and so on. We believe that future studies should further clarify the subcellular distribution of AEOL-10150, thus revealing the relevant mechanisms. With the development of clinical trials in phase 1, we will know more about the metabolism of AEOL-10150 in the human body. Basic research will further reveal the subcellular distribution characteristics of AEOL-10150, revealing its active mechanism more profoundly.

## Abbreviations

4-HNE: 4-hydroxynonenal
ALS: Amyotrophic lateral sclerosis
ARS: Acute radiation syndrome
BAL: Bronchial alveolar lavage
BARDA: Biomedical Advanced Research and Development Authority
CEES: 2-Chloroethyl ethyl sulfide
Cl_2_: Chlorine
COPD: Chronic obstructive pulmonary disease
ECE-1: Endothelin-1
ET-1: Endothelin-1
FDA: Food and Drug Administration
HSP: Heat shock protein
MCAO: Middle cerebral artery occlusion
MIP-2: Macrophage inflammatory protein-2
MPO: Myeloperoxidase
NIH: National Institutes of Health
OE: Olfactory epithelium
PARP: Poly ADP-ribose polymerase
PBA: Phenylbutyric acid
RT: Radiotherapy
SM: Sulfur mustard
SNS: Strategic National Stockpile
SOD: Superoxide dismutase
